# Comparative Study on the Antioxidant and Anti-*Toxoplasma* Activities of Vanillin and Its Resorcinarene Derivative

**DOI:** 10.3390/molecules19055898

**Published:** 2014-05-07

**Authors:** Claudio B. S. Oliveira, Ywlliane S. R. Meurer, Marianne G. Oliveira, Wendy M. T. Q. Medeiros, Francisco O. N. Silva, Ana C. F. Brito, Daniel de L. Pontes, Valter F. Andrade-Neto

**Affiliations:** 1 Laboratory of Malaria and Toxoplasmosis Biology/LABMAT, Department of Microbiology and Parasitology, Bioscience Center, Federal University of Rio Grande do Norte, Av. Salgado Filho, s/n, Lagoa Nova, Natal/RN, CEP 59000-000, Brazil; 2 Memory Studies Laboratory, Physiology Department, Bioscience Center, Federal University of Rio Grande do Norte, Av. Salgado Filho, s/n, Lagoa Nova, Natal/RN, CEP 59000-000, Brazil; 3 Laboratory of Coordination Chemistry and Polymers, Institute of Chemistry, Federal University of Rio Grande do Norte, Av. Salgado Filho, s/n, Lagoa Nova, Natal/RN, CEP 59000-000, Brazil

**Keywords:** vanillin, resorcinarene, antioxidant, anti-*Toxoplasma*

## Abstract

A resorcinarene derivative of vanillin, resvan, was synthesized and characterized by spectroscopic techniques. We measured the cytotoxicity (*in vivo* and *in vitro*), antioxidant and anti-*Toxoplasma* activities of vanillin and the resorcinarene compound. Here we show that vanillin has a dose-dependent behavior with IC_50_ of 645 µg/mL through an *in vitro* cytotoxicity assay. However, we could not observe any cytotoxic response at higher concentrations of resvan (IC_50_ > 2,000 µg/mL). The *in vivo* acute toxicity assays of vanillin and resvan exhibited a significant safety margin indicated by a lack of systemic and behavioral toxicity up to 300 mg/kg during the first 30 min, 24 h or 14 days after administration. The obtained derivative showed greater antioxidative activity (84.9%) when comparing to vanillin (19.4%) at 1,000 μg/mL. In addition, vanillin presents anti-*Toxoplasma* activity, while resvan does not show that feature. Our findings suggest that this particular derivative has an efficient antioxidant activity and a negligible cytotoxic effect, making it a potential target for further biological investigations.

## 1. Introduction

*Toxoplasma gondii* is an intracellular protozoan of the phylum Apicomplexa. It is one of the most widespread parasites in Nature, which can infect a wide range of host species, including one-third of the World’s human population [1]. As an opportunistic human pathogen, *T. gondii* may cause severe disease in immunocompromised individuals, such as HIV positive patients, and congenitally infected newborns [2]. In these patients, upon reactivation of a latent infection, this parasite can cause retinochoroiditis or CNS lesions [3,4].

Infection can occur congenitally or it can be acquired through any of the forms of the parasite, such as oocysts ingested from contaminated food with cat feces and across cysts in undercooked meat. In addition, it can also be transmitted through acute maternal infection during pregnancy [5,6].

Nowadays, toxoplasmosis is being treated with a combination of sulfadiazine and pyrimethamine, both key enzyme inhibitors in the biosynthesis of pyrimidines in *T. gondii* [7]. However, these treatments may cause adverse reactions, including suppression of the bone marrow, teratogenic effects in the first trimester of pregnancy and sulfadiazine hypersensitivity [5,8,9]. Thus, there is an increasing need for new drugs and treatment regimens against toxoplasmosis. Manipulations of natural products mainly derived from plants and insects might be a good choice in order to diminish the damage caused by conventional treatment or even improve treatment outcome.

Cultural knowledge about medicinal plants handling represent a vital role in the discovery of novel natural products with chemotherapeutic properties [10,11,12,13,14,15]. The use of medicinal herbs in developed countries kept its popularity due to historical and cultural reasons even with the access to modern medicine for most of the population [16]. On the other hand, in the developing countries, 65%–80% of the population depends exclusively on the medicinal plants for basic healthcare. It corresponds to 80% of the population in Africa, 71% in Chile, 40% in Colombia and 80% in Brazil [17,18]. 

**Figure 1:**
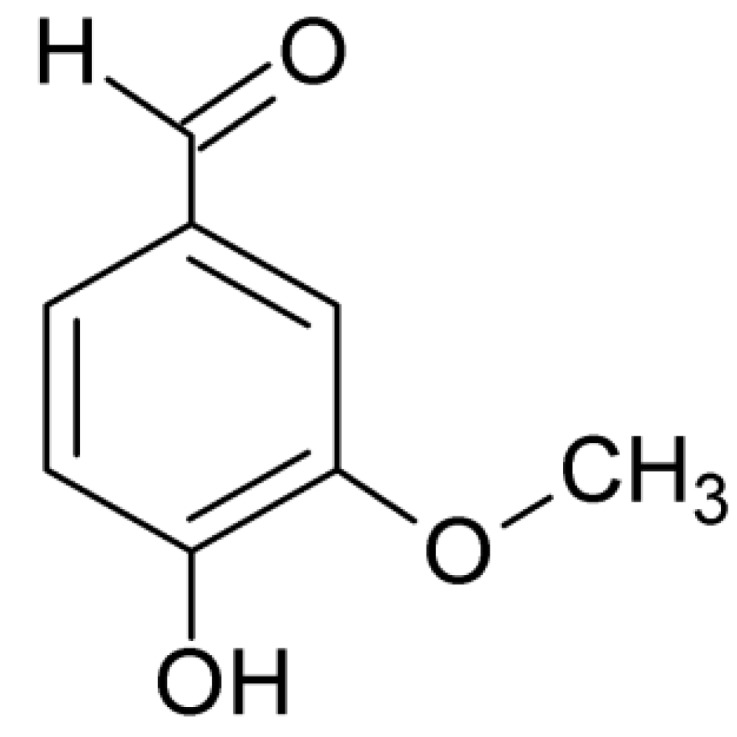
Vanillin structure.

Thereby, vanillin (4-hydroxy-3-methoxybenzaldehyde, Figure 1), a compound isolated from the bean and pod of tropical vanilla orchid is widely used in the food and beverage industry and is responsible for the characteristic vanilla flavor. This substance is also relevant for the synthesis of different agrochemicals, antifoaming and pharmaceutical products [19]. 

Besides its industrial and food application this compound has been the subject of several scientific investigations in the last years, such as the identification of antioxidant properties [20], antimicrobial activity [21,22,23] as well as antimutagenic [24,25] and anticarcinogenic actions [26]. Conversely, vanillin may also induce oxidative stress in yeast cells [27].

Part of these biological properties can be attributed to the fact that vanillin is a phenolic compound. The antioxidant activity of phenols is attributed to their ability to scavenge free radicals. Other important phenol compounds include the dihydroxybenzenes, and its derivatives [28].

Indeed, vanillin presents different functional groups, including ether and aldehyde moieties, besides the phenolic group. The presence of a methoxy group adjacent to the phenol hydroxyl gives origin to a group of compounds isolated from plants called vanilloids, which includes substances such as vanillin, eugenol and capsaicin [29].

**Figure 2:**
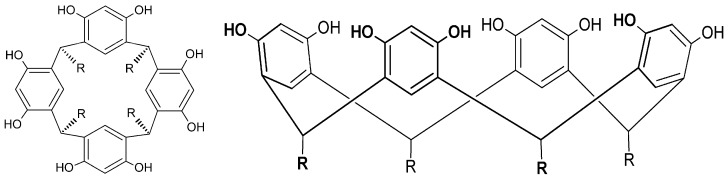
Resorcinarene general structure.

The presence of the aldehyde functional group turns possible the synthesis of vanillin derivatives by condensation reactions. It is known from the works of Niederl and Vogel [30] that the reaction of aldehyde with resorcinol (1,3-dihydroxybenzene) produces macrocyclic structures called resorcinarenes (Figure 2). These structures generally adopt a bowl or vase-like conformation characterized by the presence of a wide upper rim (resorcinol hydroxyl groups) and a narrow lower rim (R groups from the starting aldehydes). This class of compounds is particularly interesting because the structural organization and the presence of moieties with different polarities: polar region due the eight hydroxyl groups in the upper rim and nonpolar region originated by the benzene rings.

**Scheme 1:**
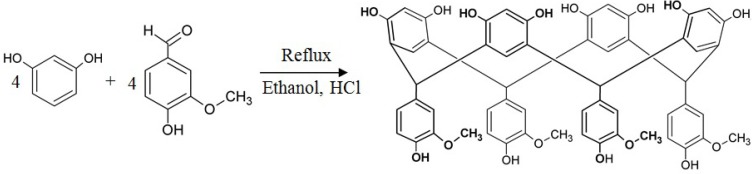
Resvan synthesis scheme.

The condensation of vanillin and resorcinol (Scheme 1) enables the preparation of the compound C-4-hydroxy-3-methoxyphenylcalix[4]resorcinarene, here identified as resvan (Figure 2), with a large number of phenolic groups. 

Our interest in studying substances isolated from plants and its derivatives, and knowledge of antiprotozoal activity of the vanilloids compounds [31] led us to examine the antioxidant properties of the resorcinarene synthesized from vanillin and to evaluate the toxicity *in vivo* and *in vitro* and the antioxidant and the anti-*Toxoplasma* activity of this compound compared to vanillin.

## 2. Results and Discussion

### 2.1. Infrared Analysis

The assignments of the signals observed in the infrared (IR) vibrational spectra of resvan were made in a qualitative form by comparison with similar data reported in the literature [32] for related compounds and with the signals observed in the vibrational spectra of the starting materials vanillin and resorcinol (Table 1).

**Table 1:** Infrared spectra and assignments of the main bands (cm^−1^) of the compounds resorcinol, vanillin and resvan.

Assignment	Resorcinol	Vanillin	Resvan
νO-H (phenol)	3447	3447	3411
νC-H (aromatic)	3012	3013	3008
νC-H (aldehyde)		2855	
νC=O (aldehyde)		1666	
νC-C (benzene)	1616	1596	1616 and 1518
νCH_3_ (methoxy)		1463	1463
δCH (methine bridge)			1430
νC-CHO (aldehyde)		1265	
νC-O (ether)		1200	1204
δCH_3_ (methoxy)		1172	
νC-OH	1150	1155	1154
δCCH (in-plane)		1123	1123
νO-CH_3_		1028	1030
δC-C-CHO (out-of-plane)		732	
δC-C=O (in-plane)		588	

The IR spectrum of the compound has a broad absorption at 3411 cm^−1^ related to phenolic hydroxyl stretching (νOH) of the upper and lower rim. Absorptions at 1616 and 1518 cm^−1^ are assigned to C-C stretching of the aromatic rings present in the structure. As resvan formation involves the condensation of the vanillin aldehyde group with resorcinol, the infrared peaks related to the aldehyde functional group are no longer expected in the IR spectrum. This result was confirmed since the aldehyde bands at 1666 (νC=O), 1267(νC-CHO), 734 (δC-CO out-of-plane) and 590 cm^−1^ (δC-CO in-plane) present in the vanillin IR spectrum were not observed in the resorcinarene spectrum [33]. On the other hand, the IR retains the peaks related to the methoxy group also observed in vanillin, however with a slight variation in wavenumber. The peak corresponding to the methine carbon of the macrocyclic structure was observed at 1430 cm^−1^.

### 2.2. Electronic Spectra Analysis

**Figure 3:**
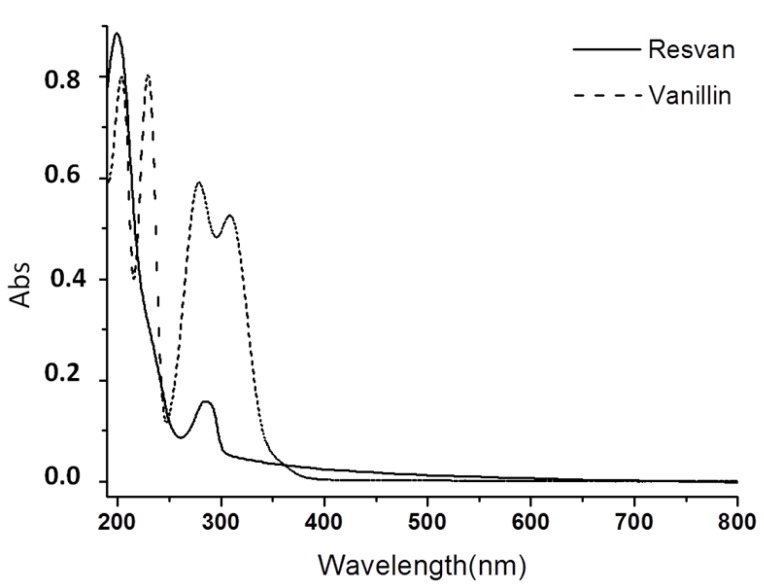
Electronic spectrum in water of resvan (5.5 × 10^−5 ^mol L^−1^) and vanillin (5.8 × 10^−5^ mol L^−1^).

The electronic spectrum of the resvan in water solution presented two bands at 198 (ε = 1.7 × 10^4^ M^−1^ cm^−1^) and 288 nm (ε = 3.5 × 10^3^ M^−1^ cm^−1^), besides two shoulders around 233 nm and 316 nm (Figure 3). Spectra of benzene derivatives usually have at least three bands assigned to π-π* electronic transitions in the UV-Vis region. The resorcinol spectrum shows these bands at 194, 218 and 272 nm. Such bands are shifted around 10 nm to longer wavelength in the vanillin spectrum due to the presence of different chemical groups bonded to the benzene ring. For this substance these bands were observed at 204, 229 and 279 nm. Additionally the presence of a fourth band at 309 nm assigned to n-π* transitions involving the carbonyl group of the aldehyde substituent can be verified.

The resvan electronic spectrum had a similar trend showing bands in the ultraviolet region at 198, 233 and 288 nm, all assigned to π-π* transitions, and a shoulder at approximately 316 nm that extends to the visible region assigned to n-π* transitions. 

### 2.3. Citotoxicity and Acute Toxicity

**Figure 4:**
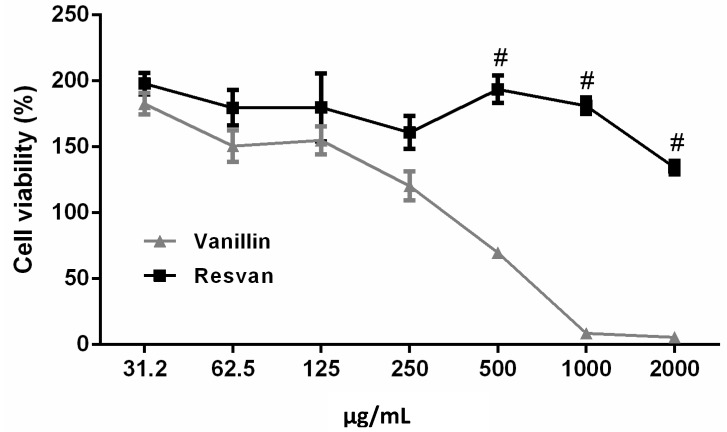
Influence of vanillin and resorcinarene in RAW 264.7 macrophage cell viability after treatment for 24 h. Results are expressed in percentage of control. Three independent experiments are performed in triplicate. (^#^ *p* < 0.001 compared to 500, 1,000 and 2,000 µg/mLvanillin treatment).

The cytotoxicity assay with murine macrophage cells was performed to compare the effect of vanillin and its derivative molecule, resvan (Figure 4). In the cytotoxicity assay analysis, the *T-*test revealed significant differences between resvan and vanillin treatment (t = 2.692, *p* = 0.0192). In the multiple *T*-test, differences between vanillin and resvan treatment were detected for 500, 1,000 and 2,000 µg/mL concentrations. In this test vanillin presented an IC_50_ of 645 µg/mL. In other studies, vanillin shows a dose-dependent activity, where a high concentration induces a reduction of 50% in cell viability, including tumoral cells [26]. However, no cytotoxic effects were observed when cells were treated with different concentrations of resvan (IC_50_ > 2,000 µg/mL), a vanillin derivative. The low cytotoxicity found for vanillin *in vitro* corroborates the results obtained by other authors for vanillin and the *o-*vanillin isomer [26,34]. The cytotoxicity results obtained suggest compounds vanillin and resvan can be used safely in other tests in order to investigate their biological activity. 

The results suggest better biological activity and altered toxicity properties after molecular alterations in the vanillin structure. Thus, modifications seem to be promising for other biological and chemical studies. The acute toxicity *in vivo* studies of vanillin and resvan were performed based on OECD guidelines, with modifications [35], where both exhibited a significant safety margin as indicated by a lack of systemic and behavioral toxicity up to 500 mg/kg, that is, no related adverse effects for animals treated with 500 mg/kg during first 30 min, 24 h and even up to 14 days after administration of chemicals. Thus, the result indicates that resvan had no significant impact regarding the *in vivo* toxicity when compared to vanillin. Also, no adverse effects were observed in the control group receiving DMSO 8% (data not shown). 

The divergent toxicity (*in vivo* and *in vitro* models) presented by vanillin, in relation to resvan, suggests a different metabolic route for this compound. Resvan was nontoxic in both *in vivo* and *in vitro* evaluation. This result provides further evidence of involvement of the liver in biotransformation the vanillin into a nontoxic metabolite.

### 2.4. Antioxidant Activity

**Figure 5:**
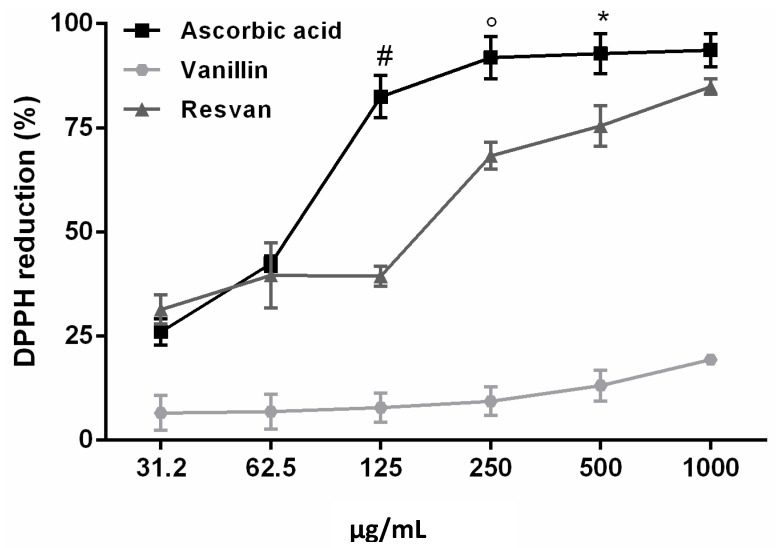
Antioxidant activity of ascorbic acid, vanillin and resvan assessed by DPPH radical scavenging activities method (^#^ *p* < 0.001 compared to 125 µg/mL Ascorbic acid treatment; ° *p* < 0.01 compared to 500 µg/mL ascorbic acid treatment; * *p* < 0.05 compared to 1,000 µg/mL ascorbic acid treatment).

The DPPH method was used to evaluate the antioxidant activity of vanillin and its resorcinarene derivative. It is an appropriate method when radical scavenging abilities of compounds are investigated at room temperature. The measure is widely recognized for this purpose and presents high correlation with other methods such as ferric reducing power and the total phenolics content [36,37]. The results suggest that resvan presented an excellent free radical scavenging activity, 84.9%, when compared to vanillin, 19.4%. The resvan activity was similar to that observed for the ascorbic acid, 93.6%, used as a standard product for the assay (Figure 5).

The reducing property indicates that the antioxidant compounds are electron donors and can reduce the oxidized intermediates of the lipid peroxidation process, so that they can act as primary and secondary antioxidants [36].

The reduction of DPPH as indicated is followed by monitoring the decrease in its absorbance at a characteristic wavelength during the reaction. In its radical form, DPPH absorbs at 517 nm, but upon reduction by an antioxidant (AH) or a radical species (Re), the absorption disappears [37]. From the results obtained in the present study, it is evident that the interaction of a potential antioxidant with DPPH* depends on its structural conformation. Certain compounds react very rapidly with the DPPH reducing the number of DPPH molecules which corresponding to the number of available hydroxyl groups. However, for the majority of the compounds tested the mechanism seems to be more complex [38,39].

In this paper, the results corroborate to those obtained in other studies [37,40,41]. Here, have attempted to explain the results obtained using the DPPH method for the compound as well as ascorbic acid. Even if antioxidant activity is extremely beneficial for scavenging free radicals, it’s even more important in cell signaling and in defense against pathogens, an can be very harmful as they react with several important molecules, altering the structure and function of proteins and initiating the process of oxidation of fatty acids, which are the cell membrane lipids [42]. 

This higher antioxidant activity found in resvan is extremely relevant especially when compared to vanillin, already recognized as an antioxidant substance [20,43] and resvan has more activity. This effect is possibly due to the higher number of phenolic hydroxyls found in resvan (Figure 2) compared to vanillin (Figure 1). These phenolic hydroxyls probably act in scavenging of the free radicals. Another advantage of this result is that the resvan, beyond its improved antioxidant activity, also showed lower toxicity for cultured macrophages. Hence, the structural change generated an efficient less toxic and more active compound. These encouraging data were expected considering that the chemical structure of resvan presents several hydroxyl groups, which could act in the clearance of free radicals in the DPPH assay.

### 2.5. Determination of in Vivo Antiprotozoal Activity of the Compounds

**Figure 6:**
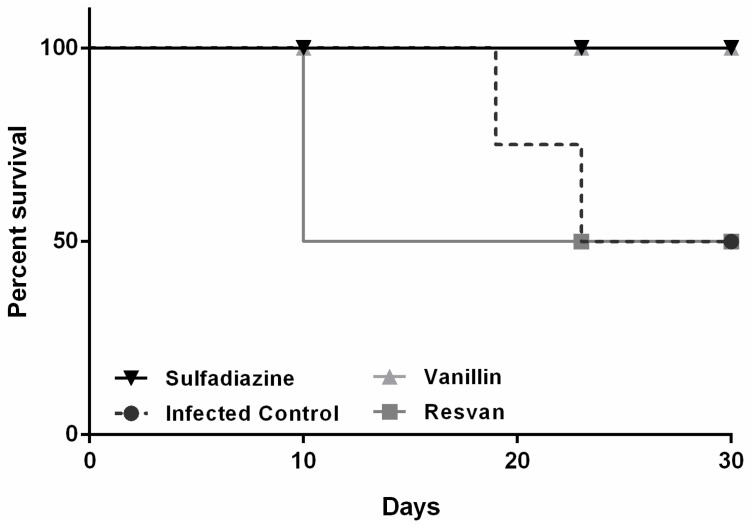
Survival curves of Swiss female mice after infection with the ME49 strain of *T. gondii* and treated with sulfadiazine (200 mg/Kg), resvan (500 mg/Kg) or vanillin (500 mg/Kg).

**Figure 7:**
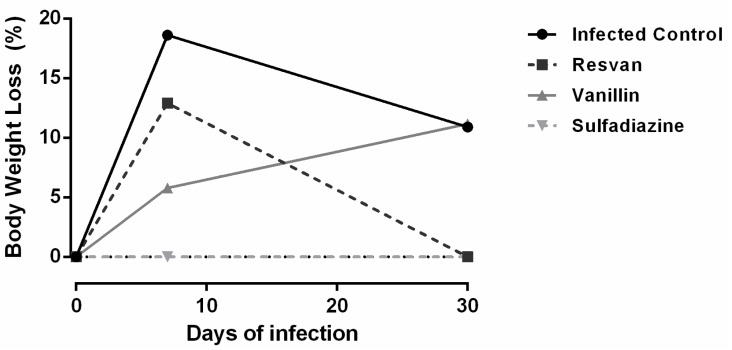
Change in body weight in Swiss mice infected with 25 cysts of *T. gondii* ME49 strain and treated with sulfadiazine (200 mg/Kg), resvan (500 mg/Kg) or vanillin (500 mg/Kg).

Mice were infected with *T. gondii* ME49 strain and treated for six consecutive days. The 30-day observation period showed that mortality only occurred in the infected and untreated control groups (19th and 23th days) and in mice treated orally with resvan (10th day). The groups of animals treated with vanillin and sulfadiazine displayed a higher survival trend compared to the untreated control group (Figure 6), evidencing the antitoxoplasmic activity of these regimens. Vanillin also caused a reduction in loss body weight in mice, indicating also its contribution to reduce the disease morbidity (Figure 7).

**Figure 8:**
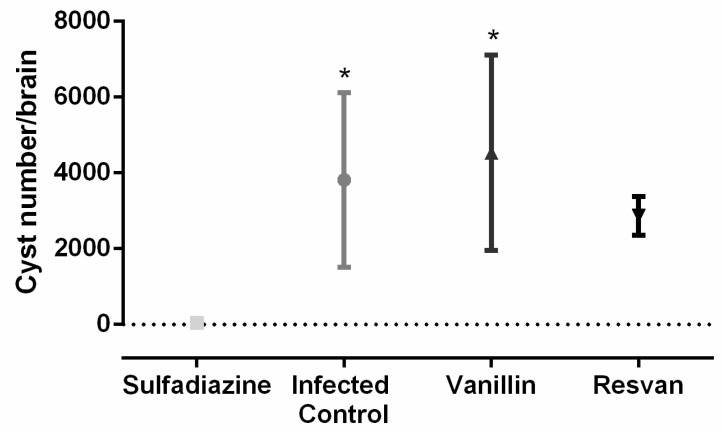
Number of brain cysts in Swiss mice infected with 25 cysts of *T. gondii* ME49 strain and treated with sulfadiazine (200 mg/Kg), resvan (500 mg/Kg) or vanillin (500 mg/Kg) (* *p* < 0.05 compared to sulfadiazine treatment).

The survival time for the vanillin and sulfadiazine groups was significantly longer (*p* < 0.05) compared to the infected control group. Treatment of the mice with vanillin significantly increased their survival rate on the 30th day after challenge by approximately 50% compared with the infected control group (*p* < 0.05). These data are similar to those observed with *Artemisia annua* tea infusion [44], which produced a similar effect to that achieved with the reference drug, sulfadiazine, in reducing morbidity (Figure 8) and mortality (Figure 7), but in this work brains cysts were increased in groups treated with vanillin‒4,533 cysts, compared to sulfadiazine treatment group‒4 cysts (Figure 8). For the number of cysts in mice brain, one way ANOVA analysis revealed significant differences between treatment groups [F(3,12) = 5.084, *p* = 0.0168). *Post hoc* analysis revealed an increased number of cysts in mice brains for infected control and vanillin treatments when compared with sulfadiazine treatment. However, no differences between sulfadizine and resvan treatment were detected by the *Bonferroni’s pos hoc* test . The ME49 strain is a better alternative in *in vivo* antitoxoplasmic activity tests, since it is less virulent, facilitating the chronic phase of infection [45], thereby allowing more time to assess different treatment regimes.

The importance of vanillin in inhibiting the mortality in mice with toxoplasmosis is remarkable. However, it is important to remark that, in the surviving animals, there was an increase in the number of brain cysts, probably due formation of a parasite-stressed environment, important to cyst formation [46,47,48,49]. The lower therapeutic efficiency for resvan may be due its larger antioxidant activity, which can inhibit the formation of free radicals essential for the destruction of the parasite [50]. There are no studies that report the action of vanillin or resvan in control of toxoplasmosis, which is unprecedented. There are papers that report several biological activities for vanillin and its derivatives [20,21,22,23,26,27,43], however this anti-protozoan activity was only just recently reported among vanilloid compounds [29]. Further studies to assess the antiparasitic effects *in vitro* are required.

## 3. Experimental

### 3.1. Chemicals

Vanillin and resorcinol were purchased from Vetec Química Fina Ltda – Sigma-Aldrich Corporation (Rio de Janeiro, Brazil). MTT [3-(4,5-dimethyl-2-thiazolyl)-2,5-diphenyl-2*H*-tetrazolium bromide)] and DPPH (2,2-diphenyl-1-picrylhydrazyl) were purchased from Sigma-Aldrich (St. Louis, MO, USA). All reagents were used without further purification. Other trivial chemicals used were all of analytical grade. Reaction progress was monitored by TLC using Fluka silica gel HF254.

### 3.2. Synthesis of C-4-Hydroxy-3-Methoxyphenylcalix [4] Resorcinarene (Resvan)

The compound was prepared in one-step reaction pathway (Scheme 1) using a similar procedure to that reported by Rose [51]. Vanillin (0.766 g, 5 mmol) and resorcinol (0.552 g, 5 mmol) were dissolved in ethanol (30 mL) and to the resulting solution concentrated hydrochloric acid (0.5 mL) was added dropwise with continuous stirring. The solution was kept 15 h under reflux and the beige precipitate obtained was filtered, washed with ethanol and dried. Yield: 75%.

### 3.3. Apparatus

Electronic spectra in the ultraviolet and visible (UV-Vis) regions were acquired with a Shimadzu model UV 1800 spectrophotometer using water as solvent. Infrared spectra were obtained in KBr pellets with a Thermo Nicolet Nexus 470 FT-IR spectrophotometer. When necessary (MTT Method) the optical density (OD) at 570 nm was determined in a Uv-Vis spectrophotometer (Thermoplate).

### 3.4. Cytotoxicity Assay

For the cytotoxicity assay, an adapted methodology from Tempone [52] was used. RAW 264.7 cells were cultured in Dulbecco’s modified Eagle’s medium (DMEM; GIBCO Inc., NY, USA) supplemented with 40 mg/L of gentamicin and 10% fetal bovine serum (FBS; GIBCO Inc., NY, USA). The cells were incubated in an atmosphere of 5% CO_2 _at 37 °C and were sub-cultured every 5 days. 

RAW 264.7 murine macrophage cells were seeded at 1 × 10^4^ cells per well in 96-well microplates and incubated at 37 °C for 24 h in the presence of the compounds, dissolved previously in DMSO at a concentration lower than 1% and twofold serial diluted with DMEM medium to the concentration between 31.2 µg/mL and 2,000 µg/mL. The microplates were incubated for 24 h at 37 °C in a 5% CO_2 _humidified incubator. Control cells were incubated in the presence of dimethylsulfoxide (DMSO) without drugs (1%). 

The viability of the macrophages was determined with the Diphenyltetrazolium (MTT) assay. Initially, MTT (0.5 mg/mL) was dissolved in Phosphate Buffered Saline (PBS) and sterilized through 0.22 µm membranes, and then 100 µL/well was added to a 96-well plate and left at 37 °C for 3 h. Formazan extraction was performed with DMSO (200 µL/well) at 25 °C, and the (OD) at 570 nm was determined in a Uv-Vis spectrophotometer. 

### 3.5. Animals

Swiss-Webster albino (6–8 weeks of age) were used for the toxicity and anti-*Toxoplasma* tests and received water and food *ad libitum.* *In vivo* tests were performed using Guidelines for Ethical Conduct in The Care and Use of Animals from the Federal University of Rio Grande do Norte (CEUA, Protocol number 43/2010).

### 3.6. Acute Toxicity

Acute toxicity studies were performed for the compounds following, with modifications, OECD guidelines [35]. Female Swiss mice of 8–12 weeks weighing around 23–25 g were used. Chemicals were orally administered to the animals in a dose of 500 mg/kg. The animals were observed continuously for 2 h for any symptoms of toxicity (piloerection, weight loss and postural abnormalities) and /or death. They were under observation for further 2 weeks.

### 3.7. Scavenging Activity on 1,1-Diphenyl-2-picrylhydrazyl (DPPH) Radicals

Scavenging activity on 1,1-diphenyl-2-picrylhydrazyl (DPPH) radicals by vanillin and resvan was measured according to the method described by Ye [53], with adaptations, in which decrease in absorbance of reaction mixture containing DPPH and compounds were measured after fixed time interval.

Accordingly, 100 µL of vanillin, resvan and ascorbic acid in different concentrations (31.25 µg/mL up to 1,000 µg/mL) were plated with 200 µL of DPPH solution (0.7 mM) in each well. The microplates were incubated for 30 min in room temperature and then the absorbance was measured at 517 nm in a Uv-Vis spectrophotometer. 

Percent scavenger was calculated as follows:

% S = [1 − (A1/A0)] × (100%)

where A1 corresponds to the absorbance of the samples and A0 is the absorbance of the control.

### 3.8. Determination of in Vivo Antiprotozoal Activity of the Compounds

*In vivo* tests were performed with female Swiss mice (*n* = 4), as previously described [44]. Initially mice were infected orally with 25 cysts. After 24 h, animals received orally their respectively treatment with a daily dose of vanillin (diluted in distilled water), resvan (500 mg/kg, diluted in DMSO 8%) or sulfadiazine (200 mg/kg) for 6 days after infection. The animals were observed daily for mortality and morbidity. All surviving animals were euthanized at thirty days of infection for the quantification of the parasite burden in brain tissues.

### 3.9. Statistical Analysis

Data were analysed by Multiple *T*-test or one way analysis of variance (ANOVA) followed *Bonferroni’s pos hoc* test when appropriate. The Mantel-Cox log-rank test was performed for survival time analysis using GraphPad Prism version 6.0 for Windows (GraphPad Software, La Jolla, CA, USA). 

## 4. Conclusions

Thus, we can conclude that the chemical modification proposal was efficient in increasing the antioxidant and reduce cytotoxicity, but just vanillin presented anti-*Toxoplasma* activity. These results show this derivative attractive as an efficient antioxidant with negligible cytotoxic effects and potentially interesting for further biological trials. We also highlight the importance of the vanillin acting to combat parasite infections.
